# Successful Pregnancy Outcome in an Operated Case of Budd-Chiari Syndrome Having Fetal Growth Restriction: A Twisted Tale of Gravid

**DOI:** 10.7759/cureus.42745

**Published:** 2023-07-31

**Authors:** Jatin Gupta, Kamlesh Chaudhari, Apoorva Dave, Mounica B

**Affiliations:** 1 Obstetrics and Gynaecology, Jawaharlal Nehru Medical College, Datta Meghe Institute of Higher Education & Research, Wardha, IND

**Keywords:** multidisciplinary approach, tipss, anticoagulants, foetal growth restriction, budd-chiari syndrome

## Abstract

Budd-Chiari Syndrome (BCS) primarily affects women in the reproductive age group, with an ever-increasing incidence in the general population. Due to its rarity, it is not known precisely how a pregnancy progresses in a woman with BCS and what can happen to the baby. A rare condition known as Budd-Chiari syndrome causes the hepatic veins in the liver to constrict and become blocked. The challenges in pregnancy, such as decreasing hepatic function, a rise in thrombotic and bleeding events, or ascites, have historically made pregnancy inappropriate in these people. Here, we present a case of an unbooked 24-year-old female, a known case of treated BCS with 36 weeks and three days gestation period. She was referred from a peripheral hospital to our hospital's emergency department because of having fetal growth restriction. By presenting this rare case, we expect more extensive studies will be conducted on the effect of pregnancy on BCS and the effect of BCS on pregnancy which will help obstetricians to turn this rare possibility of conception into a fair possibility.

## Introduction

Budd-Chiari syndrome (BCS), a rare but fatal condition, can cause the hepatic veins responsible for draining blood from the liver to constrict or get clogged. As a result of impaired blood flow to the liver, it might result in liver damage, liver failure, and other detrimental effects. With the Incidence of 1/1,00,000-5,00,000 in the general population, BCS mainly affects women in the reproductive age group [1]. As a result of its rarity, there is no evidence of how exactly a pregnancy progresses in a woman with BCS and what the outcome can be. Since BCS is primarily a vascular condition, a hypercoagulable state is always present, making it more challenging to deal with during pregnancy as pregnancy is already hypercoagulable. Here we report a case of a primigravida with Gestational age of 36 weeks and three days, a known case of Budd Chiari Syndrome having a Transjugular intrahepatic portosystemic shunt (TIPSS) catheter in situ and having fetal growth restriction ended with an uneventful pregnancy outcome.

## Case presentation

**Figure 1:**
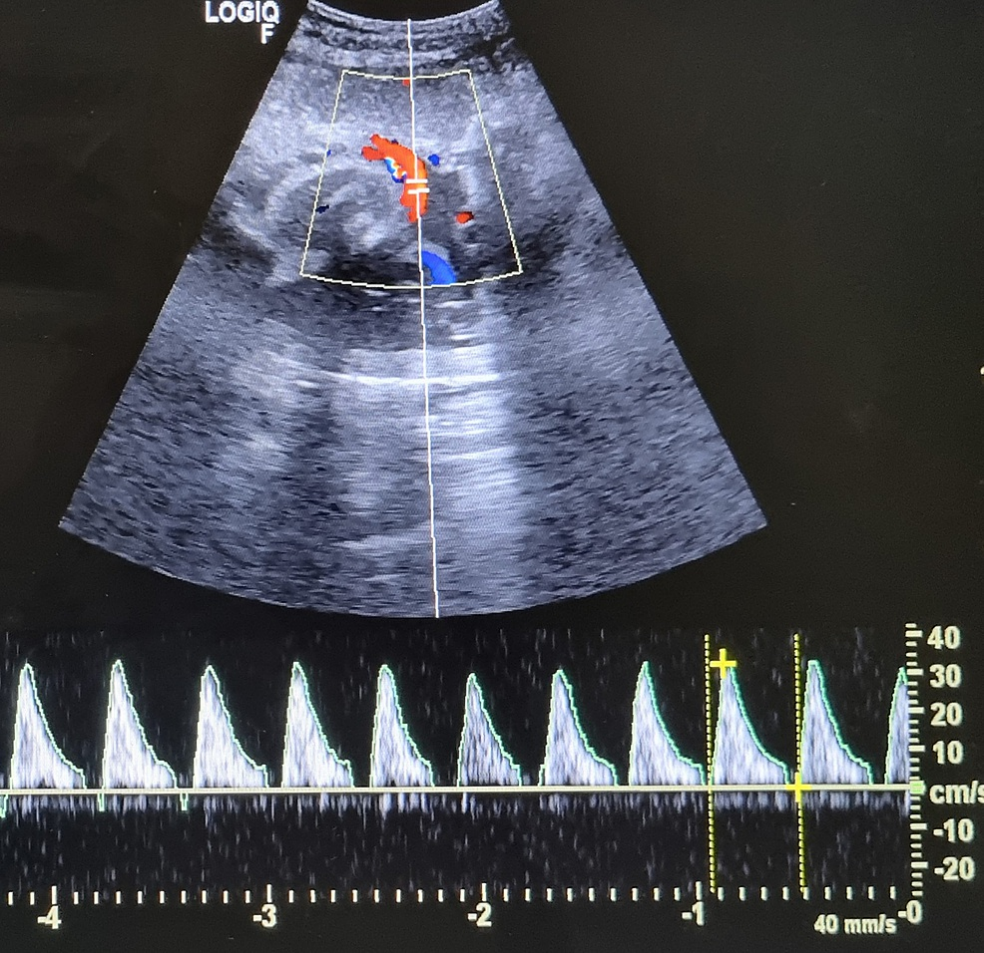
Ultrasonography Showing Doppler Abnormalities in the Form of Absent Diastolic Flow in the Umbilical Artery

An unbooked 24-year-old female, a known case of treated BCS with 36 weeks three days gestational period, was referred from the peripheral hospital to our hospital's emergency room because of having fetal growth restriction. Upon taking history, she was a postoperative case of Budd-Chiari Syndrome (BCS) with a history of portal vein thrombosis; she had undergone a Transjugular intrahepatic portosystemic shunt (TIPSS) four years back in our hospital. She conceived spontaneously, and according to the patient, her previous antenatal visits in the peripheral center were uneventful. Her latest growth scan on the day of her visit to our hospital showed Doppler abnormalities in the form of absent diastolic flow in the umbilical artery, as shown in Figure 1.

Per abdominal examination, the uterus size corresponds to 34 weeks of gestation, relaxed, cephalic presentation, head 3/5th palpable, Fetal heart sound (FHS) present, and regular 150-160 beats per minute. The liver and spleen were not palpable. Antenatal investigations were conducted, including a hemogram (Table 1), coagulation profile (Table 2), liver function test (Table 3), and kidney function test (Table 4).

**Table 1:** Hemogram MCHC: mean corpuscular hemoglobin concentration, MCV: mean corpuscular volume, MCH: mean corpuscular hemoglobin, RBC: red blood cell, WBC: white blood cell, RDW: red cell distribution width, gm: gram, pg: picogram, fl: femtolitre, microL: microlitre, mm: millimetre, %: percentage.

PARAMETERS	PATIENT VALUES	REFERENCE RANGE
Hemoglobin (gm%)	12	12-16
MCHC (%)	33.4	32-36
MCV (fl)	79.3	76-96
MCH (pg)	26.5	27-32
Total RBC Count (million/mm3)	4.54	4.5-6.0
Total WBC Count (/microL)	11500	4500-11000
Total Platelet Count (lakh/microL)	1.44	1.5-4.0
Monocytes (%)	03	2-8
Granulocytes (%)	75	50-70
Lymphocytes (%)	20	20-40
RDW (%)	15.5	12.2-16.1
Eosinophils (cells/microL)	02	0-500
Basophils	00	0-300

**Table 2:** Coagulation Profile APTT: activated partial thromboplastin time, INR: international normalised ratio.

PARAMETERS	PATIENT VALUES	REFERENCE RANGE
APTT-Control (seconds)	29.5	21-35
APTT-Patient (seconds)	38.0	21-35
Prothrombin Time-Control (seconds)	11.9	10-13
Prothrombin Time-Patient (seconds)	12.6	10-13
INR	1.06	1.0

**Table 3:** Liver Function Test ALP: alkaline phosphatase, ALT: alanine transaminase, AST: aspartate transaminase, u/l: unit per litre, mg: miligram, dl: decilitre.

PARAMETERS	PATIENT VALUES	REFERENCE RANGE
ALP (u/l)	167	44-147
ALT (u/l)	29	<40
AST (u/l)	29	<40
Total Bilirubin (mg/dl)	0.4	0.2-1.3

**Table 4:** Kidney Function Test mg: miligram, dl: decilitre, l: litre, meq: milliequivalent, mmoles: millimoles.

PARAMETERS	PATIENT VALUES	REFERENCE RANGE
Urea (mg/dl)	19	5-20
Creatinine (mg/dl)	0.6	0.7-1.3
Sodium (mmoles/l)	140	136-145
Potassium (meq/l)	4.7	3.5-5.2

**Figure 2:**
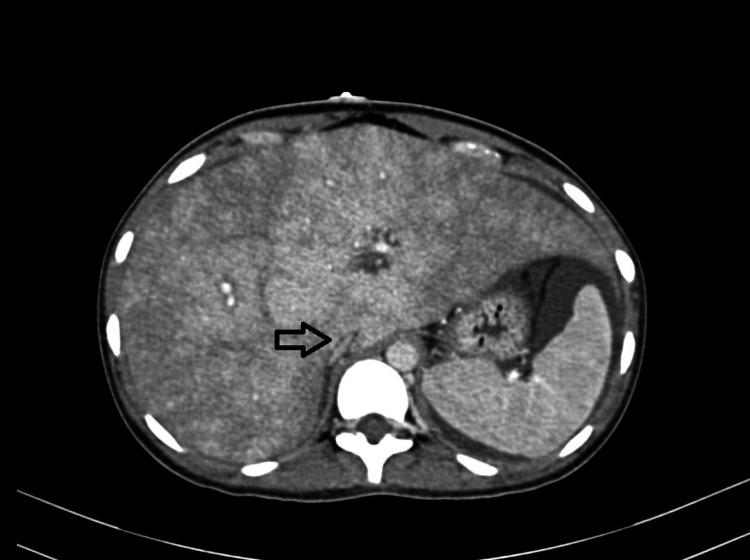
Axial Contrast-Enhanced Computed Tomography Showing Inhomogeneous Mottled Liver and Narrowing of the Intrahepatic Portion of the Inferior Vena Cava as Shown by the Pointing Arrow.

**Figure 3:**
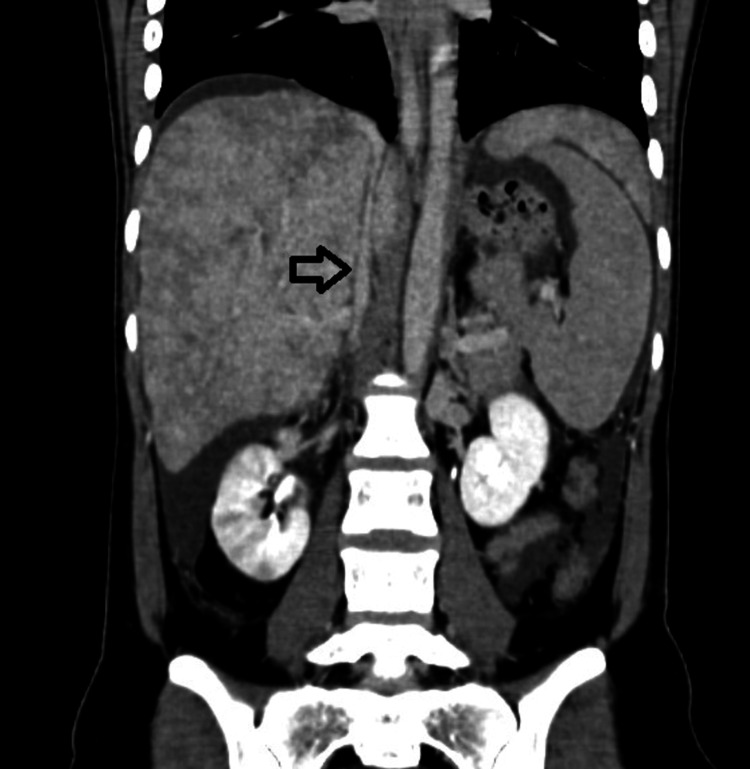
Coronal Section of Contrast-Enhanced Computed Tomography Showing Narrowing of Intrahepatic Portion of Inferior Vena Cava as Shown by Pointing Arrow.

The patient's history revealed the development of ascites four years ago, leading her to initially consult a general physician and later get referred to a gastroenterologist. She was diagnosed with Budd-Chiari Syndrome on a CT scan, as shown in Figures 2-3.

**Figure 4:**
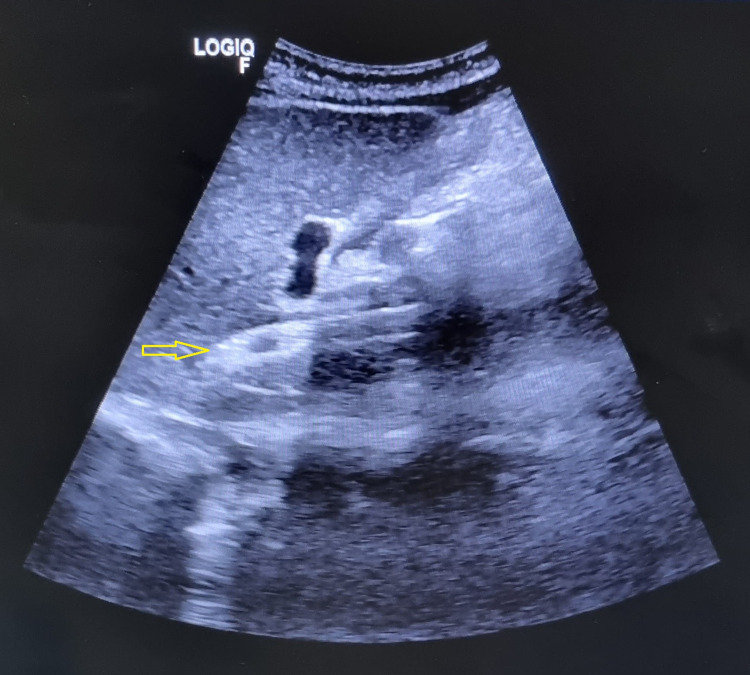
Ultrasound Upper Abdomen Showing TIPSS Stent in Situ with Monophasic Flow on Doppler

Her CECT interpretation revealed evidence of an inhomogeneous mottled liver and narrowing of the intrahepatic portion of the Inferior Vena Cava. Hepatic veins were not visualized separately. The report concluded as Budd-Chiari Syndrome and was managed with a TIPSS procedure in a tertiary care hospital. She was kept in the medical intensive care unit (MICU) for five days after the procedure. She was on oral anticoagulant medication (Tablet Nicoumalone 3 mg OD) and Tablet Aspirin 150 mg OD for the next two years. She was on observation for two weeks and was discharged once stable. In her current admission, she underwent an ultrasound of the abdomen for the maternal liver, and a repeat obstetrics ultrasound was performed. On Ultrasound of the upper abdomen, the TIPSS stent was seen in situ with the monophasic flow on Doppler, as shown in Figure 4.

Ultrasonography on admission suggested a single intrauterine live fetus appropriate to gestational age 33.4 weeks, corresponding to a weight of 2200 grams. Amniotic fluid index: 7-8 cm (oligohydramnios) with absent end diastolic flow in the umbilical artery. On upper abdomen ultrasound, multiple collaterals at porta-hepatis were noted-portal vein 14 mm, dilated, however usual in flow, TIPSS catheter in situ. A 1.3 X 1.6 cm hemangioma was noted in the right lobe of the liver. Gallbladder and liver normal, spleen of 12.4 cm size indicates borderline splenomegaly. Bilateral kidneys are normal. The patient's high-risk consent has been obtained, and a multidisciplinary opinion was taken from a medicine consultant, gastroenterologist, and interventional radiologist.

The gastroenterologist suggested that the patient can be taken for lower segment cesarean section (LSCS). The patient is then posted for emergency lower segment cesarean section because of absent diastolic flow in the uterine artery. An experienced anesthetist and obstetrician were involved in the management. Induction to delivery time was shortened under spinal anesthesia. The surgeon tried to minimize the surgery time and prevent blood loss (blood loss=300ml). LSCS was done by the Joel Cohen technique. The uterus was closed by the double-layer technique. The incision to closure time was just 18 minutes. The intraoperative period remained uneventful. There were no ascites, and there were no increased collateral vessels in the abdominal wall and pelvis. The female newborn cried immediately after birth, weighed 1.9 kg, and had an APGAR score of 9/10, which was normal, and had no complications. In the postoperative period, opioid analgesics were avoided, and NSAIDs were given. The patient was kept in the medical intensive care unit for 24 hours for observation and vital charting. Early ambulation was done, and dehydration was prevented to avoid thrombosis. She had thrombocytopenia on a post-op day one and was started on low molecular weight heparin (Enoxaparin Sodium injection, 60 mg subcutaneous BD) from the next day, which was continued for ten days. She also started taking aspirin 150 mg OD after seven days. Neonate was kept for observation in the neonatal intensive care unit (NICU) as the mother was in MICU. Paladai feeds were given to the baby. Afterward, the mother was shifted to the postnatal ward, and the baby was moved to the mother's side.

## Discussion

Budd-Chiari Syndrome (BCS) is a rare vascular condition with hepatomegaly, ascites, and abdominal pain as its clinical hallmarks. It is an unusual illness brought on by thrombotic or non-thrombotic obstruction (due to narrowing) of the hepatic venous outflow. The block may occur anywhere along the venous channel, from the hepatic venules to the inferior vena cava (IVC) junction and right atrium [2]. Usually, patients with BCS cannot conceive (infertility is high in BCS) [3]. Between 3.8 and 21.5% of pregnant and postpartum women have BCS; however, its rarity makes it unknown how frequently a woman with BCS becomes pregnant [4]. Although infertility is frequent with BCS, childbearing is safe if timely medical intervention and proper care are provided to the mother. Endovascular intervention and good post-surgical care can obtain improved fertility and pregnancy outcomes. Due to the substantial risk associated with Budd-Chiari syndrome and pregnancy, there is a debate. Pregnancy has historically been contraindicated in these individuals due to the difficulties it entails, such as worsening hepatic function, an increase in thrombotic and bleeding events, or ascites. Patients with stable illness also have a higher chance of recurring postpartum thrombosis, necessitating more surgical procedures such as angioplasty, TIPSS, or portosystemic bypass [5]. The transhepatic intrajugular portosystemic shunt (TIPSS) procedure succeeded in treating most cases of Budd-Chiari syndrome to a great extent. TIPSS is a useful therapeutic option for BCS patients, particularly those not candidates for liver transplantation [6]. For a successful pregnancy outcome in a patient with a known case of Budd-Chiari Syndrome, proper antenatal care, timely hospitalization, regular monitoring and investigations, patient compliance, prompt and timely diagnosis, and treatment are required. Managing a case of pregnancy in BCS can be challenging and require an integrated approach toward the patient.

Ksheerasagar S et al. [1] reported a case of pregnancy with BCS, stating that "it is challenging to manage a case of pregnancy in BCS Close monitoring of the patient, adequate anticoagulation, fetal growth monitoring, and liver status along with a multidisciplinary team (MDT) is required to have successful outcomes." Merz et al. [7] reported a case of successful pregnancy outcome in Budd-Chiari Syndrome which stated that "In order to have a favorable outcome of pregnancy in women with BCS, an interdisciplinary team of specialists in gastroenterology, radiology, visceral surgery, medicine, obstetrician, and neonatology was involved." Richa Subhash Udhwani et al. [8] reported a case of BCS (due to IVC web) complicating the pregnancy in a patient with poor obstetric history. They stated that a prompt early diagnosis, good history, and imaging are essential to properly manage pregnancy in BCS. Faisal Khan et al. [9], in their study on pregnancy outcomes in the case of BCS, suggested that pregnancy results in patients treated for BCS can be favorable if it is beyond 20 weeks of gestation and managed adequately. Pulmonary hypertension is an important finding that requires further attention. These patients should be addressed in hospitals experienced in managing high-risk pregnancies.

Management includes regular follow-ups with a senior physician, gastroenterologist, interventional radiologist, and obstetrician. Any abnormality in fetal Doppler or maternal complications like a deranged coagulation profile, liver disease, or intrapartum complications can indicate emergency cesarean section. The duration of the surgery should be minimized intraoperatively to decrease postoperative complications due to increased bleeding. To achieve an uneventful postoperative period, the patient should be managed in the intensive care unit. Intravenous fluids are indicated to prevent thrombosis due to dehydration. Early ambulation and therapeutic Enoxaparin Sodium injection, which is low molecular weight heparin (60 mg subcutaneously BD), is recommended after consulting an expert physician [10].

Pasquale Martinelli et al. [11] reported a case of pregnancy in a woman with BCS, bicornuate uterus, and protein C deficiency treated with a portosystemic shunt. They stated that there is a requirement for proper pre-conceptional counseling in women with BCS, including monitoring of liver and vascular pathology, antithrombotic medications for prophylaxis (if thrombophilic abnormalities coexist), and searching for other co-morbidities which can affect the pregnancy. The mother was delivered at 29 weeks of gestation due to severe ascites and a deranged coagulation profile, but there was fetal demise due to prematurity. In contrast, the maternal condition was stable in our case, she had no ascites, and adequate precautions were taken to avoid fetomaternal adverse outcomes. Folch BF et al. [5] stated that not all patients with BCS can be allowed to conceive. So, an experienced obstetrician must individualize each case preconceptionally on whether to allow conception.

## Conclusions

There are few cases in the medical literature related to pregnancy in Budd-Chiari Syndrome (BCS). Most of the time, there are challenges for the mother to get conceived and carry a pregnancy. There are very few pregnancies that have successful outcomes when it comes to maternal and newborn morbidity and mortality. Hence, establishing the impact of Budd-Chiari Syndrome on pregnancy and vice versa is of utmost importance. With an interdisciplinary approach and optimization of patient conditions, it is possible to have favorable outcomes in managing antenatal patients with BCS. Further, more extensive studies are required regarding this entity to give a better guideline for better-managing pregnancy in Budd-Chiari Syndrome.
